# Invasion of Host Cells by Microsporidia

**DOI:** 10.3389/fmicb.2020.00172

**Published:** 2020-02-18

**Authors:** Bing Han, Peter M. Takvorian, Louis M. Weiss

**Affiliations:** ^1^Department of Pathology, Albert Einstein College of Medicine, New York, NY, United States; ^2^Department of Pathogenic Biology, School of Basic Medical Sciences, Shandong University, Jinan, China; ^3^Department of Biological Sciences, Rutgers University, Newark, NJ, United States; ^4^Department of Medicine, Albert Einstein College of Medicine, New York, NY, United States

**Keywords:** microsporidia, invasion apparatus, polar tube, spore wall, sporoplasm, cell–host interaction

## Abstract

Microsporidia are found worldwide and both vertebrates and invertebrates can serve as hosts for these organisms. While microsporidiosis in humans can occur in both immune competent and immune compromised hosts, it has most often been seen in the immune suppressed population, e.g., patients with advanced HIV infection, patients who have had organ transplantation, those undergoing chemotherapy, or patients using other immune suppressive agents. Infection can be associated with either focal infection in a specific organ (e.g., keratoconjunctivitis, cerebritis, or hepatitis) or with disseminated disease. The most common presentation of microsporidiosis being gastrointestinal infection with chronic diarrhea and wasting syndrome. In the setting of advanced HIV infection or other cases of profound immune deficiency microsporidiosis can be extremely debilitating and carries a significant mortality risk. Microsporidia are transmitted as spores which invade host cells by a specialized invasion apparatus the polar tube (PT). This review summarizes recent studies that have provided information on the composition of the spore wall and PT, as well as insights into the mechanism of invasion and interaction of the PT and spore wall with host cells during infection.

## Introduction

Microsporidia are a diverse group of unicellular obligate intracellular spore-forming eukaryote parasites that were identified more than 150 years ago with the identification of *Nosema bombycis* (Naegeli, 1857) as the etiologic agent of Pébrine (pepper disease) in silkworms. Microsporidia are widely distributed in nature and there are over 200 genera and 1400 species which have been characterized (Cali et al., 2017). Phylogenetic analysis of microsporidia have demonstrated that they are related to the Fungi, either as a basal branch of the Fungi or as a sister group (Weiss et al., 1998; Lee et al., 2008; Capella-Gutiérrez et al., 2012), and that they are most likely related to the Cryptomycota (Corsaro et al., 2014; Keeling, 2014).

As parasites, they can infect a wide variety of hosts ranging from invertebrates to vertebrates and have been reported from every major group of animals from protists to mammals, including man. They can be found environmentally in terrestrial, marine, and freshwater ecosystems (Cali and Takvorian, 2004). Infection by microsporidia in economically important invertebrate hosts such as silkworm, honeybee, and shrimp as well as vertebrates such as fish can cause significant economic losses (Stentiford et al., 2016). Microsporidia infections in daphnia, nematode, locust, honeybee, and mosquito play important roles in the regulation of the population size of their hosts (Brambilla, 1983; Higes et al., 2010; Pan et al., 2018).

There are multiple routes of transmission for microsporidia to spread in nature. The most common of these being vertical transmission (the direct transfer of infection from parent to progeny) and horizontal transmission (the transmission of the pathogens from one individual to another of the same generation by oral transmission of spores through contaminated food and water) (Steinhaus and Martignoni, 1970; Fine, 1975; Goertz et al., 2007; Becnel et al., 2014). In humans the majority of infections by microsporidia are thought to be zoonotic and transmitted by the ingestion of spores in food or water (Fayer and Santin, 2014).

Since the 1980s, microsporidia have been identified as significant opportunistic parasites of humans (Cali and Owen, 1988; Weber et al., 1994; Didier and Weiss, 2011; Weiss and Becnel, 2014) with only a few reports prior to that time (Strano et al., 1976). Currently, 9 genera and 17 species have been reported to infect humans (Weiss and Becnel, 2014). Microsporidia are important pathogens in patients with advanced AIDS, bone marrow transplantation, organ transplantation, and patients using new antibody based immune modulatory agents (Didier and Khan, 2014). Infection is also being increasingly recognized in the elderly and pediatric population as well as travelers (Gumbo et al., 1999; Ghoshal et al., 2015).

**FIGURE 1:**
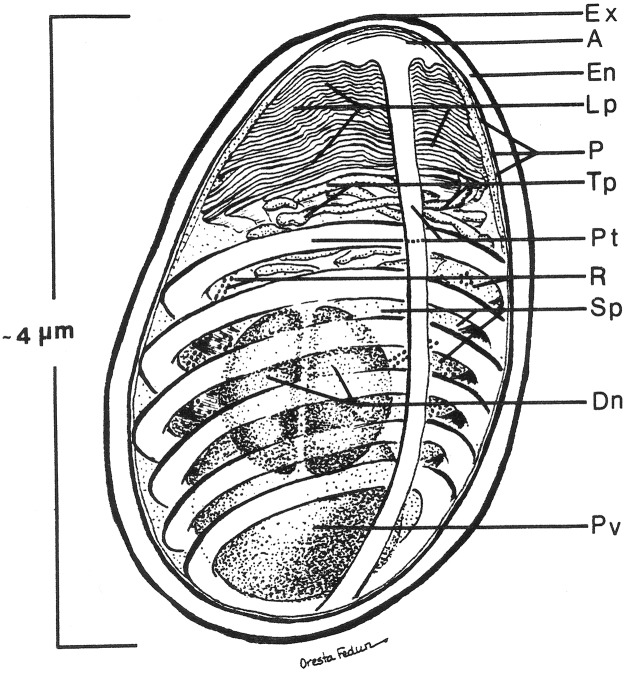
Diagram of the internal structure of a microsporidian spore. The spore coat has an outer electron dense region called the exospore (Ex) and an inner thicker electron lucent region, endospore (En). A unit membrane (P) separates the spore coat from the spore contents. The extrusion apparatus, anchoring disc (A), polar tubule (Pt), lamellar polaroplast (lp), and tubular polaroplast (Tp) dominate the spore contents and is diagnostic for microsporidian identification. The posterior vacuole (Pv) is a membrane-bound vesicle that sometimes contains a “membrane whirl” or “glomerular like” structure or flocculent material or some combination of these structures. The spore cytoplasm is dense and contains ribosomes (R) in tightly coiled helical array. The nucleation may consist of a single nucleus or a pair of abutted nuclei, diplokaryon (D). The size of the spore depends on the particular species and can vary from less than one micron to over 10 microns. The number of polar tubule coils is also variable from a few to thirty or more, again depending on the species. Reprinted with the permission of the publisher (Cali and Takvorian, 2014).

While Microsporidia are a diverse group of unicellular parasites, they all form a diagnostic spore containing a coiled polar filament surrounding the nucleus or diplokaryon and its associated cytoplasmic organelles, the sporoplasm (Figure 1). The resistant spore can persist in the environment for months and in some cases, for years under the right conditions (Kramer, 1970). This highly resistant spore is the only microsporidial form that is extracellular and is the infective stage (Vavra and Larsson, 1999, 2014; Cali and Takvorian, 2014). The spores of microsporidia are generally small, oval- or pyriform- shaped, resistant structures that vary in length from approximately 1 to 12 μm (Sprague and Vavra, 1977; Canning and Lom, 1986; Olson et al., 1994). Those infecting mammals are generally 1 to 4 μm in length (Bryan et al., 1991; Weber et al., 1994).

The typical mature microsporidian spore has an electron- dense outer spore coat overlying an inner thicker lucent coat followed by a membrane surrounding the spore contents. Diagnostic for the microsporidia is a polar filament, anteriorly attached to an anchoring disk (AD) with the straight part of the polar filament immediately following and encompassed by a membranous sheath. Projecting from the anterior portion of the sheath are a series of tightly packed array of membrane, the lamellar polaroplast, which is followed by clusters of wider tubules, the tubular polaroplast. The central portion of the spore contains a nucleus or pair of abutted nuclei (diplokaryon), in cytoplasm with tightly packed ribosomes. The posterior of most spores contain a highly variable structure referred to as the posterior vacuole. Surrounding the nuclear and cytoplasmic central region of the spore is the coiled polar filament [i.e., polar tube (PT)]. There may be few to many dozens of cross sections of the polar filament coil, arranged in a single or multiple rows, depending on the organism (Cali and Takvorian, 2014).

**FIGURE 2:**
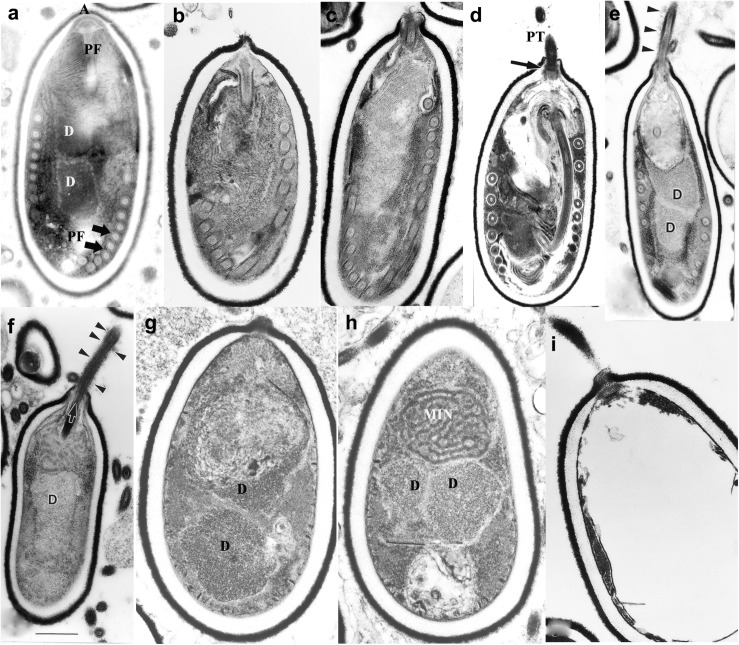
Germination of microsporidian spore. *Anncaliia algerae* spores incubated in germination buffer and processed for TEM. The sequence of images illustrates the events that occur in the germination process. **(a)** Typical *A. algerae* spore; **(b)** spore coat bulging; **(c)** spore coat rupture and polaroplast expanded; **(d)** early eversion and translocation of polar tube (PT); **(e)** majority of PT extruded, nuclear and cytoplasmic structures still in spore; **(f)** no PT coils remain in spore but sporoplasm still present; **(g)** spore “membrane channels” visible immediately below endospore; **(h)** posterior vacuole, diplokaryon and MIN (sporoplasm) the last structures exiting the spore shell; **(i)** empty spore shell with PT still attached. Reprinted with the permission of the publisher (Cali and Takvorian, 2014).

Microsporidia infect host cells by employing a unique, highly specialized invasion process that involves the spore wall (SW), PT, and the infectious sporoplasm (SP). This germination event which results in the transfer of the infective sporoplasm into a susceptible host cell requires a series of complex events, which include environmental changes necessary to activate the spore (Leitch et al., 1993; Leitch and Ceballos, 2008). An activated spore undergoes a progression of changes to both the spore coat and spore contents (Figure 2). An initial change consists of a bulge of the apical end of the spore accompanied by a narrowing of the endospore coat in that region. The apical attachment complex of the polar filament, its associated membranes, and the filament proper of the inactive spore, all become reoriented upon activation. Additionally, the apical complex everts, forming a collar-like structure as the polar filament, now termed the PT exits from the spore-wall (Cali et al., 2002; Takvorian et al., 2005; Cali and Takvorian, 2014). The extruded PT serves as a conduit for the sporoplasm to transfer from the spore into a new host (Cali et al., 2002; Cali and Takvorian, 2014; Takvorian et al., 2019). Non-activated spores may also be phagocytized by a host cell and eventually discharge their PTs, depositing the sporoplasm into the host cytoplasm (Franzen, 2004, 2005; Franzen et al., 2005). On occasion, discharged sporoplasms have been observed interacting with the host cell plasmalemma and being taken into the cell by endocytosis/phagocytosis (Takvorian et al., 2013).

**FIGURE 3:**
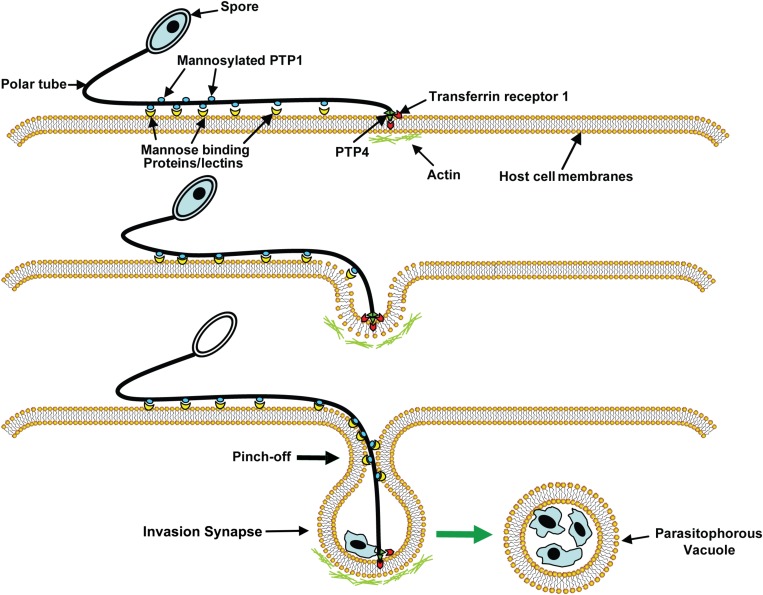
A model of microsporidia invasion of a host cell. Polar tube protein 1 (PTP1) interacts with mannose binding proteins (MBPs) on the host surface adhering the PT to the host surface allowing the PT to form an invasion synapse by pushing into the host cell membrane. Interactions of PTP1 (and possibly PTP4) with the host cell membrane in the invasion synapse exclude the external environment from the invasion synapse creating a protected microenvironment for the extruded microsporidian sporoplasm. Polar tube protein 4 (PTP4) epitopes at the tip of PT interact with Transferrin receptor 1 (TfR1) or other host cell interacting proteins (HCIPs) on the host cell surface triggering signaling events such as clathrin-mediated endocytosis and the involvement of host cell actin in the final invasion event with formation of a parasitophorous vacuole. Reprinted with permission of the publisher (Han et al., 2017).

The PT upon discharge then interacts with the host cell forming an invagination in the host cell membrane, thereby creating a microenvironment, which we have termed the invasion synapse (Figure 3). The proteins [polar tube proteins (PTPs), sporoplasm surface proteins, and host cell receptors] that participate in the formation of the invasion synapse remain to be fully characterized. Within this protected microenvironment, the sporoplasm which has traveled down the PT into this synapse is delivered to the host cell and invasion occurs (Han et al., 2017). The exact mechanism of entry of microsporidia into their host cells is unknown. It is possible that the PT either pierces the host cell membrane in this synapse delivering the sporoplasm directly into the host cells, or that the sporoplasm itself may interact with the host cell membrane during invasion (Takvorian et al., 2013; Han et al., 2017). Based on observations on the Encephalitozoonidae (Han et al., 2017, 2019), we hypothesize that that the sporoplasm interacts with the host cell membrane and an invasion vacuole is formed (Figure 3). Once the infectious sporoplasm enters the host cell it undergoes development into meronts (proliferative forms), sporonts, sporoblasts (developing spore) and finally mature spores (Visvesvara, 2002).

## Spore Wall Proteins (SWPs)

The spore wall contains three layers: an electron-dense, proteinaceous exospore, an electron lucent endospore, and an underlying plasma membrane (Vávra, 1976; Canning and Lom, 1986; Cali and Owen, 1988). This spore wall maintains the morphology of the spore and protects the organism from harsh environmental conditions before it infects the host (Shadduck and Polley, 1978). It has been demonstrated that the spore wall contains chitin as well as numerous spore wall proteins (SWPs) (Vávra, 1976). In addition, to protecting the spore from the environment, the spore wall also interacts with the environment and host cell and is involved in the process of activating PT germination (Yang et al., 2018). SWPs that localize to the exospore are exposed directly to the host cells and environment. These SWPs are in all probability involved in the process of host cell binding, signaling, or enzymatic interactions (Hayman et al., 2005; Southern et al., 2007). For example, some SWPs have been demonstrated to bind to mucin and proteoglycans (Hayman et al., 2005; Southern et al., 2007), which would enable spores to bind the mucin layer in the gastrointestinal tract, thereby, facilitating invasion of intestinal epithelial cells by the PT on germination (Weiss et al., 2014). Endosporal SWPs are also in all likelihood involved in the processes of the endospore formation, PT interaction, and spore germination.

According to several studies on the composition of the spore wall, there are multiple SWPs present in both the exospore and endospore (Table 1). The identification of these SWPs has primarily focused on the Encephalitozoonidae, *Encephalitozoon cuniculi* (Ec), *E. hellem* (Eh) and *E. intestinalis* (Ei), which infect humans as well as other mammalian hosts, and *Nosema bombycis* (Nb), which can infect silkworms (Yang et al., 2018). Several SWPs have been identified from the Encephalitozoonidae of which EcSWP1, EiSWP1, EiSWP2, EhSWP1a, and EhSWP1b are localized to exospore and EcEnP1, EiEnP1, EcEnP2, EcSWP3 and EcCDA which are localized to the endospore (Bohne et al., 2000; Hayman et al., 2001; Brosson et al., 2005; Peuvel-Fanget et al., 2006; Xu et al., 2006; Southern et al., 2007). With the availability of genome data for the Encephalitozoonidae as well as many other microsporidia on MicrosporidiadB.org (part of EuPathdB.org) homologs of these SWPs have been found in most of the Encephalitozoonidae as well as in the other microsporidia genomes found on MicrosporidiadB (such as microsporidia that infect invertebrates) (Weiss et al., 2014). Examples of such homologs include, *Antonospora locustae* SWP2 (AlocSWP2) and *Enterocytozoon hepatopenaei* SWP1 (EHSWP1) (Chen et al., 2017; Jaroenlak et al., 2018).

**TABLE 1:** The identified spore wall proteins of Microsporidia.

	Protein	Subcellular location	Function domain	Mw (kDa)	Amino acids/GenBank ID	References
*Encephalitozoon cuniculi*	EcSWP1	Exospore	–	45.9	450 aa ECU10_1660	Bohne et al., 2000
	EcEnP1	Endospore	HBM	40.6	357 aa ECU01_0820	Peuvel-Fanget et al., 2006
	EcEnP2/EcSWP3	Endospore	Transmembrane	22.5	221 aa ECU01_1270	Peuvel-Fanget et al., 2006; Xu et al., 2006
	EcCDA	Endospore and plasma membrane	Glycoside hydrolase and deacetylase	28.1	254 aa ECU11_0510	Brosson et al., 2005
*Encephalitozoon intestinalis*	EiSWP1	Exospore	–	41.5	388 aa AF355750.1	Hayman et al., 2001
	EiSWP2	Exospore	–	107.2	1002 aa AF355749.1	Hayman et al., 2001
	EiEnP1	Exospore and endospore and polar membrane layer	HBM	39.1	348 aa EF539266	Southern et al., 2007
*Encephalitozoon hellem*	EhSWP1a	Exospore	–	54.9	509 aa FJ870923	Polonais et al., 2010
	EhSWP1b	Exospore	–	57.9	533 aa FJ870924	Polonais et al., 2010
*Nosema bombycis*	NbSWP5	Endospore and polar tube	–	20.3	186 aa EF683105	Li et al., 2012
	NbSWP7	Exospore and endospore	–	32.8	287 aa EOB13707.1	Yang et al., 2015
	NbSWP9	Exospore, endospore and polar tube	Transmembrane helix region (TMHMM)	42.8	367 aa EOB13793.1	Yang et al., 2015
	NbSWP11	Exospore and endospore	DnaJ domain	52.3	446 aa EF683111	Yang et al., 2014
	NbSWP12	Exospore and endospore	BAR-2 domain	26.6	228 aa EF683112	Chen et al., 2013
	NbSWP16	Exospore	HBM	44.0	221 aa EOB14338	Wang et al., 2015
	NbSWP25	Endospore	HBM	30.7	268 aa EF683102	Wu et al., 2009
	NbSWP26	Exospore, endospore and plasma membrane	HBM	25.7	223 aa EU677842	Li et al., 2009
	NbSWP30	Endospore	–	32.1	278 aa EF683101	Wu et al., 2008
	NbSWP32	Exospore	–	37.4	316 aa EF683103	Wu et al., 2008
	EOB14572	Endospore and polar tube	Four tandem repeats	37.0	316 aa NBO_24g0018	Wang et al., 2017
*Enterocytozoon hepatopenaei*	EHSWP1	Exospore and endospore	HBM, BAR-2	27.0	228 aa MG015710	Jaroenlak et al., 2018
*Antonospora locustae*	AlocSWP2	Exospore and endospore	GPI, HBM	25.0	222 aa KX255658	Chen et al., 2017

*Nosema bombycis* which infects the silkworm *Bombyx mori*, has been studied as a model microsporidian for decades [since it was first identified by Louis Pasteur (Pasteur, 1870)]. Fourteen hypothetical SWPs were identified by proteomic analysis from *Nosema bombycis* (Wu et al., 2008). While some of these have homologs in the other microsporidia genomes on MicrosporidiadB.org, many of them have only been identified in *Nosema bombycis*. According to immunoelectron microscopy studies of these hypothetical SWPs, NbSWP5, NbSWP16 and NbSWP32 are located in the exospore and NbSWP25, NbSWP30, EOB14572 are located in the endospore (Wu et al., 2008, 2009; Li et al., 2012; Wang et al., 2015, 2017). NbSWP7 and NbSWP9 were found to be present in both the spore wall and PT (Yang et al., 2015, 2017). NbSWP11 was found on the membranous structures of the sporoblast and mature spore (Yang et al., 2014). NbSWP12 was located both inside and outside of the spore wall (Chen et al., 2013). NbSWP26 was expressed largely in endospore and plasma membrane during endospore development, but sparsely distributed in the exospore of mature spores (Li et al., 2009).

Chitin is the main component of the endospore, and chitin has been reported to be the major component of fibrils that form bridges across the endospore and to be part of the fibrillary system of the exospores, which is essential in maintaining spore cell structure and function (Erickson and Blanquet, 1969; Han and Weiss, 2018). The presence of chitin in the spore wall is useful as a target of diagnosis as it can be stained by fluorescence dyes such as Calcofluor white or Uvitex 2B. These fluorescent brighteners are widely used for identifying microsporidia in clinical and environmental samples (Vavra and Chalupsky, 1982; Ghosh and Weiss, 2009).

## Polar Tube Proteins (PTPs)

All microsporidial spores possess a unique, highly specialized invasion apparatus consisting of the polar filament, which coils inside of the spore and connects to a mushroom-shaped AD at the anterior end of the spore (Vávra, 1976; Takvorian and Cali, 1986). Upon appropriate environmental stimulation, the PT will be rapidly extruded from the spore and then serve as a conduit for the nucleus and sporoplasm passage into the host cell, the entire process taking less than 2 seconds (Weidner, 1972; Frixione et al., 1992). Although it has been over 125 years since the first reports of the existence of the PT by light microscopy (Thelohan, 1894), and more than 50 years since the first use of electron microscopy to image the polar filament inside of the spore (Huger, 1960), this structure, its protein composition, the mechanism of PT extrusion, and sporoplasm transport within the tube are still enigmatic.

The mechanism and chemical factors necessary for spore germination are poorly understood. Various stimuli (pH, cations, and anions) have been identified to trigger germination, some of which appear to be microsporidian species specific (Table 2). Swelling of the polaroplast and posterior vacuole presumably due to the increasing of osmotic pressure inside of the spore, probably involving aquaporins (Ghosh et al., 2006), has been observed in many microsporidia during the germination process (Thelohan, 1894; Ohshima, 1937; Lom and Vavra, 1963). A study of trehalase function in *Anncaliia (Nosema) algerae* demonstrated that the cleavage of the disaccharide trehalose into glucose by trehalase could rapidly increase the intrasporal hydrostatic pressure inside of the spore and that this increase in hydrostatic pressure could trigger spore germination (Undeen, 1990; Undeen and Meer, 1994). This increase in intrasporal osmotic pressure from the breakdown of trehalose has been postulated to induce germination in aquatic microsporidia (Undeen and Vander Meer, 1999). This may, however, not be the mechanism in other microsporidia as a study of the germination kinetics of individual *Nosema bombycis* spores, which belong to the terrestrial microsporidia, using laser tweezers Raman spectroscopy and phase-contrast microscopy imaging revealed a different germination mechanism (Miao et al., 2018). The dynamics of imaging intensity of individual spore germination detected by phase-contrast microscopy revealing that the germination speed and the germination rates are different with different germination methods, but the length of germination time was relatively constant, showing the homogeneity of *Nosema bombycis* spore germination. The actual change of intracellular macromolecules such as trehalose, nucleic acid, and protein were tracked by the single-cell Raman spectroscopy, and this demonstrated that there was no change in the intensities of trehalose peaks prior to germination, nor were new peaks of saccharides observed, indicating that spore germination in this related microsporidia is probably not due to the action of trehalase on trehalose (Miao et al., 2018).

**TABLE 2:** Conditions for activation and discharge of polar tubes.

Organism	*In vitro* method of PT germination	References
*Amblyospora* sp.	1.6M sucrose plus 0.2M KCl, pH 9	Undeen and Avery, 1984
*Edhazardia aedis*	0.1M KCl, pH 10.5	Frixione et al., 1992
*Encephalitozoon hellem*	140 mM NaCl, 5 mM KCl, 1 mM CaCl_2_, 1 mM MgCl 2, pH 9.5 or 7.5 with and without 5% H_2_O_2_	Leitch et al., 1993 He et al., 1996
*Encephalitozoon hellem*	140 mM NaCl, 1 mM CaCl_2_, 1 mM MgCl_2_, 5 mM KCl, pH 7.5 for 15min then 5% H_2_O_2_ for another 15 min	Han et al., 2019
*Encephalitozoon intestinalis*	140 mM NaCl, 5 mM KCl, 1 mM CaCl_2_, 1 mM MgCl_2_, pH 9.5 or 7.5 with and without 5% H_2_O_2_	He et al., 1996
*Encephalitozoon intestinalis*	Spores from urine resuspended in 0.025 N NaOH in phosphate buffered saline	Beckers et al., 1996
*Glugea fumiferanae*	Chlorides of alkali metal ions at pH 10.8: CsCl, RbCl, KCl, NaCl, or LiCl	Ishihara, 1967
*Glugea hertwigi*	Calcium ionophore A-23187	Weidner, 1982
	pH shift from neutral (7.0) to alkaline (9.5) in 150 mM phosphate buffer	
	50 mM sodium citrate in 100 mM glycylglycine buffer pH 9.5	
	150 mM phosphate buffer in 100 mM glycylglycine buffer pH 9.5	
*Gurleya* sp.	Dessication followed by rehydration with normal saline	Gibbs, 1953
*Nosema* sp.	3% 40-volume H_2_O_2_	Walters, 1958
*Nosema algerae*	KHCO_3_-K_2_CO_3_ buffer pH 8.8	Vavra and Undeen, 1970
*Nosema algerae*	KCl, NaCl, RbCl, CsCl, or NaF, pH 9.5; KHCO_3_, pH 9.0 (0.1 to 0.3M solutions) requires pretreatment in distilled H_2_O	Undeen, 1978
*Nosema algerae*	0. 05M halogen anion Br^–^, Cl^–^, or I^–^ in combination with Na^+^ or K^+^ pH 9.5; or 0.05M F^–^ in combination with Na^+^ or K^+^ pH 5.5	Undeen and Avery, 1988
*Nosema algerae*	0.1M NaCl buffered at pH 9.5 with 20 mM glycine-NaOH or borate-NaOH	Undeen and Avery, 1988, Undeen and Frixione, 1991
*Nosema algerae*	0.1M NaCl buffered at pH 9.5 with 20 mM Tris-borate	Frixione et al., 1992
*Nosema algerae*	Alkali metal cations in 0.1M NaCl or KCl, pH 9.5 or 0.1M NaNO_2_, pH 9.5 or Na^+^ ionophore monesin in 0.04M NaCl pH 9.5	Frixione et al., 1994
*Nosema apis*	Dehydration in air, followed by rehydration with neutral distilled H_2_O	Kramer, 1960
*Nosema apis*	Dehydration in air, followed by rehydration in phosphate buffered saline, pH 7.1	Olsen et al., 1986
*Nosema apis*	0.5M NaCl with 0.5M NaHCO_3_, pH 6	de Graaf et al., 1993
*Nosema bombycis*	30% H_2_O_2_ or 30% H_2_O_2_ with 1% NaHCO_3_	Kudo, 1918
*Nosema bombycis*	Boiled digestive fluid of silkworm or 3% H_2_O_2_	Ohshima, 1927
*Nosema bombycis*	Digestive fluid of silkworm or liver extract medium pH > 8.0	Trager, 1937
*Nosema bombycis*	NaOH (N/10 to N/160) pH 11–13 neutralized with HCl to pH 6.0–9.0	Ohshima, 1937
*Nosema bombycis*	KOH (N/7 to N/640) neutralized with HCl to pH 6.5–8.0	Ohshima, 1964a
*Nosema bombycis*	0.375M KCl, 0.05M Glycine, 0.05M KOH pH 9.4–10.0	Ohshima, 1964b
*Nosema bombycis*	1.5 to 3% H_2_O_2_	Ohshima, 1966
*Nosema bombycis*	0.1N KOH followed by preheated silkworm hemolymph	Ishihara, 1968
*Nosema bombycis*	0.05M Glycine, 0.05M KOH, and 0.375M KCl, pH 10.5	Liu et al., 2016
*Nosema costelytrae*	Pretreatment with 0.2M KCl pH 12 followed by 0.2M KCl pH 7	Malone, 1990
*Nosema heliothidis*	Pretreatment with 0.15M cation (K, Na, Li, Rb, or Cs) at pH 11, followed by 0.15M cation (K), pH 7	Undeen, 1978
*Nosema helminthorum*	Mechanical pressure	Dissanaike, 1955
*Nosema locustae*	Dehydration with 2.5M sucrose or 5% polyethylene glycol followed by 0.1M Tris-HCl, 0.1M NaCl or 0.1M glycine-NaOH, 0.1M NaCl pH 9–10	Undeen, 1990
*Nosema locustae*	Dehydration in air followed by rehydration in 0.1M Tris-HCl, pH 9.2, 37°C	Whitlock and Johnson, 1990
*Nosema michaelis*	Pretreatment in veronal acetate buffer, pH 10, followed by tissue culture medium 199	Weidner, 1972
*Nosema pulicis*	Weak acetic acid/iodine water	Korke, 1916
*Nosema whitei*	Dehydration in air, followed by rehydration with neutral distilled H_2_O	Kramer, 1960
*Nosema furnacalis*	0.17M KCl, 10 mM Na_2_EDTA, 25 mM *N,N-bis*(2-hydroxyethyl) glycine (Bicine), 30 mM glucose, pH 8.0	Sagers et al., 1996
*Perezia pyraustae*	Dehydration in air, followed by rehydration with neutral distilled H_2_O	Kramer, 1960
*Plistophora anguillarum*	0.1M Potassium citrate-HCl pH 3 to 4 or 0.01M KHCO 3-K_2_CO_3_ pH 10 or 0.5 to 50% H_2_O_2_	Hashimoto et al., 1976
*Plistophora hyphessobryconis*	5% H_2_O_2_	Lom and Vavra, 1963
*Spraguea lophii (Glugea americanus)*	pH shift from acid/neutral to alkaline (pH 9.0) in 0.5M glycylglycine or 0.5M carbonate buffer containing 2% mucin or 0.5M poly-D-glutamic acid	Pleshinger and Weidner, 1985
*Spraguea lophii (Glugea americanus)*	Calcium ionophore A-23187	Pleshinger and Weidner, 1985
*Spraguea lophii (Glugea americanus)*	Phosphate buffered saline pH 8.5–9.0 containing 0.1–0.5% porcine mucin.	Weidner et al., 1984
*Spraguea lophii (Glugea americanus)*	Storage in 0.05M Hepes, retreatment in 10^–5^ M Ca^2+^ pH 7, followed by Hepes pH 9.5 containing 2% mucin.	Weidner et al., 1995
*Thelohania californica*	Mechanical pressure	Kudo and Daniels, 1963
*Thelohania magna*	Mechanical pressure	Kudo, 1916, 1920
*Vairimorpha necatrix*	Pretreatment with 0.15M cation (K, Li, Rb, or Cs), pH 10.5, followed by 0.15M cation (Na or K), pH 9.4	Undeen, 1978
*Vairimorpha plodiae*	Pretreatment with 0.1 or 1M KCl, pH 11, followed by 0.1 or 1M KCl, pH 8.0	Malone, 1984
*Vavraia culicis*	0.2M KCl, pH 6.5 (one isolate) pH 7.0–9.0 (another isolate)	Undeen, 1983
*Vavraia oncoperae*	Pretreatment with 3 mM EDTA followed by 0.2M KCl pH 11	Malone, 1990

**FIGURE 4:**
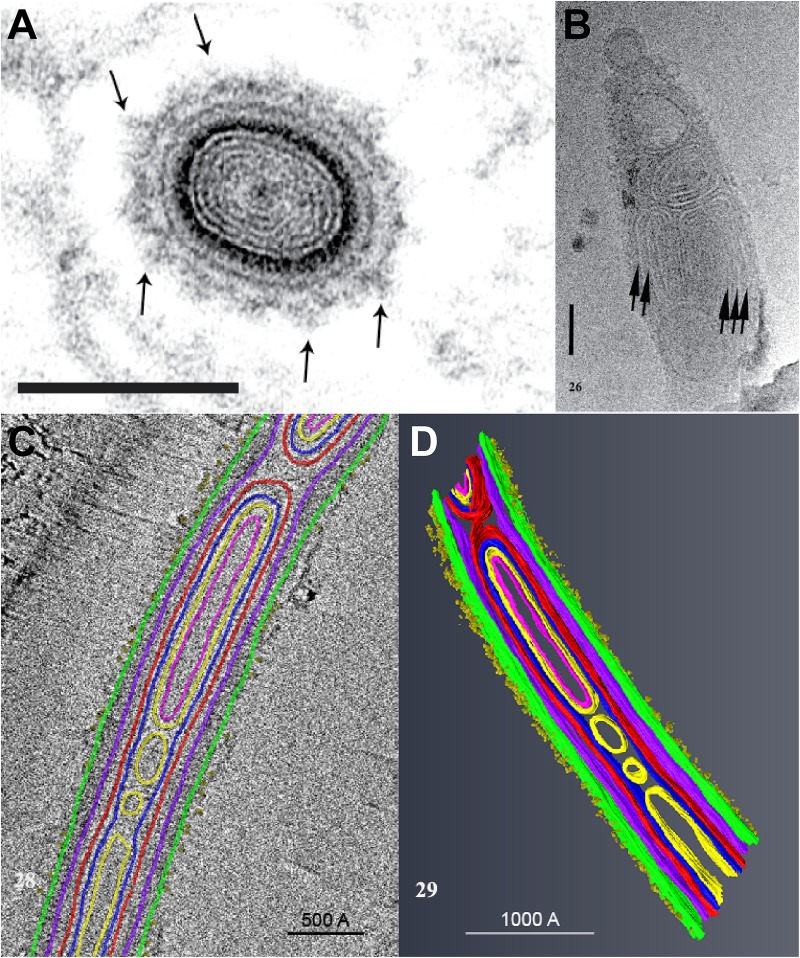
An ultrastructural study of the extruded PT of *Anncaliia algerae*. **(A)** Light and transmission electron microscopy (TEM) showed the cross section of an extruded PT from spores. The lumen of the tube is filled with about eight alternating concentric thin ED and electron lucent (EL) rings of material. The outer wall of the PT is enclosed by a relatively thick ED wall which is in turn covered by additional rings of material. The outermost PT surface is a ring of medium dense fibrous material with tufts of fibers projecting outward (arrows). Bar is 100 nm. **(B)** CTEM of the distal end of PTs showed that end of the PT containing multiple tightly packed membranes (short arrows) that will give rise to the sporoplasm membrane “terminal sac.” Note the closed tip of the tube. Bar is 50 nm. **(C)** Tomogram of a portion of PT containing cargo and membranes, and its surface is covered with tufts of fibrillar material. The different PT structures are color coded to the various densities visible in the stacks of images and identified by color. **(D)** The tomogram was segmented and 3D models generated from it using Amira software. Reprinted with the permission of the publisher (Takvorian et al., 2019).

A study using Cryo-Transmission Electron Microscopy (CTEM) to examine the structure of extruded PTs of *Anncaliia algerae* has shown that the PT is composed of various structures containing masses of tightly folded or stacked membranes (Figure 4) (Takvorian et al., 2019). This study illustrated that the “sperm head” shaped sporoplasm traverses the PT as a fully intact membrane bound cellular entity. The PT surface was shown to be covered with fine fibrillary material which was interpreted to be modified glycoproteins on the surface of PT (Figure 4A). Furthermore, the CTEM image of the PT terminus revealed that the distal end of the PT (Figure 4B), has a closed tip that can form a terminal sac before the PT tip is forced to open (Takvorian et al., 2019).

The everting PT, and the PT within the intact spore, is not empty, but has been shown by several authors to be filled with electron-dense materials (Cali et al., 2002; Vavra and Larsson, 2014; Takvorian et al., 2019) which are thought by some to be unpolymerized PTPs and perhaps membranes (Kudo and Daniels, 1963; Weidner, 1972, 1976). According to several ultrastructural observations eversion of the PT has been likened to a tube sliding within a tube (or a glove finger being turned inside out) and it has been further hypothesized that PTPs polymerize on the forming tube when they exit at the distal tip of the PT (Weidner, 1982; Weidner et al., 1995). Currently, however, there is no data demonstrating polymerization of cloned PTPs into tube-like structures.

Studies conducted to date on the composition of the PT have resulted in the identification of five distinct PTPs (Weiss et al., 2014). These studies used various Encephalitozoonidae, but genomic data from MicrosporidiadB.org indicates that these five PTPs are also found in other microsporidia such as *A. locustae, T. hominis*, and *A. algerae* (Table 3).

**TABLE 3:** Polar tube proteins PTP1 to PTP5 in microsporidia.

	PTP1	PTP2	PTP3	PTP4	PTP5
*Encephalitozoon cuniculi*	395 aa	277 aa	1256 aa	276 aa	251 aa
	ECU06_0250	ECU06_0240	ECU11_1440	ECU07_1090	ECU07_1080
*Encephalitozoon Encephalitozoon intestinalis*	371 aa	275 aa	1256 aa	279 aa	252 aa
	Eint_060150	Eint_060140	Eint_111330	Eint_071050	Eint_071040
*Encephalitozoon hellem*	453 aa	272 aa	1284 aa	278 aa	251 aa
	413 (EhATCC)	EHEL_060160	EHEL_111330	EHEL_071080	EHEL_071070
	EHEL_060170				
*Encephalitozoon romalae*	380 aa	274 aa	1254 aa	280 aa	251 aa
	EROM_060160	EROM_060150	EROM_111330	EROM_071050	EROM_071040
*Antonospora locustae*	355 aa	287 aa	Partial sequence	381 aa	242 aa
	ORF1050*	ORF1048*		ORF969*	ORF968*
		568 aa (PTP2b)			
		ORF1712*			
		599 aa (PTP2c)			
		ORF1329*			
*Paranosema grylli*	351 aa	287 aa	Partial sequence	381 aa	Partial sequence
*Enterocytozoon bieneusi*	nd	283 aa	1219 aa	nd	nd
		EBI_26400	EBI_22552		
*Trachipleistophora. hominis*	nd	291 aa	1518 aa	Partial sequence	259 aa
		THOM_1756	THOM_1479	THOM_1575	THOM_1161
*Nosema ceranae*	456 aa	275 aa	1414 aa	208 aa	268 aa
	NCER_101591	NCER_101590	NCER_100083	NCER_100526	NCER_100527
Nosema bombycis**	409 aa	277 aa	1370 aa	222 aa	271 aa
	NBO_7g0016	AEK69415	AEF33802	ACJZ01000169 (3927–4595)	ACJZ01002324 (213–1028)
*Anncaliia algerae*	407 aa	3 partial sequences	1203 aa	254 aa	240 aa
	KI0ABA33YN06FM1		KI0APB23YG12FM1	KI0ANB26YM04FM1	KI0AGA10AA09FM1
*Vittaforma cornae*	nd	293 aa	Partial sequence	254 aa	204 aa
		VICG_01748	VICG_01948	VICG_01195	VICG_01807
*Vavraia culicis floridensis*	nd	291 aa	1864 aa	372 aa	356 aa
		VCUG_00650	VCUG_02017	VCUG_02471	VCUG_02366
*Edhazardia aedis*	nd	307 aa	1447 aa	465 aa	252 aa
		EDEG_00335	EDEG_03869	EDEG_03857	EDEG_03856
			1284 aa		
			EDEG_03429		
*Nosema parisii*	nd	251 aa	1177 aa	nd	nd
		NEQG_02488	NEQG_00122		
*Octosporea bayeri*	nd	nd	Partial sequence	Partial sequence	212 aa
			ACSZ01010190	ACSZ01005588	ACSZ01000826

nd, not determined, probably because of high sequence divergence or incomplete assembly of the genome. For PTP1 there are also some differences in the number of aa for the different strains of both *E. cuniculi* and *E. hellem* (Peuvel et al., 2000). *A. locustaehttps://www.ncbi.nlm.nih.gov/assembly/GCA_007674295.1/. **Nosema bombycis (annotated sequences of *Nosema* bombycis and *Nosema antheraeae* are deposited in Genbank as the following accession numbers: ACJZ01000001-ACJZ01003558). *O.bayeri* from Broad Institute (https://microsporidiadb.org/micro/; https://www.broadinstitute.org/fungal-genome-initiative/microsporidia-genome-sequencing).

The unusual solubility properties of PTs, which resist dissociation in 1% SDS and 9M urea but dissociate in various concentrations of 2-mercaptoethanol (2-ME) or dithiothreitol (DTT), has been used to produce PTP preparations for proteomic analysis (Keohane et al., 1994, 1996). Using this approach, polar tube protein 1 (PTP1) was first isolated from microsporidia by treating glass bead disrupted spores with SDS and Urea to remove most of the proteins and then solubilizing the residual PTs with DTT. This was followed by further purification of the DTT solubilized PTs by the use of reverse-phase high-performance liquid chromatography (HPLC) (Keohane et al., 1994, 1996). Amino acid analysis of the major protein that was purified, named PTP1, demonstrated that it is proline rich, which would contribute to the high tensile strength and elasticity of PTP1. These properties are probably important for the discharge and passage of sporoplasm through the PT (Keohane et al., 1996, 1998; Delbac et al., 2001). Further analysis of PTP1 demonstrated that it is a mannosylated protein with a significant number of O-linked mannosylation modification sites which make it possible for PTP1 to interact with mannose binding receptors on the surface of host cells and enables the PT to bind to the cell surface during infection (Xu et al., 2003, 2004; Bouzahzah and Weiss, 2010; Bouzahzah et al., 2010). Interestingly, PTP1 has been found to be quite divergent in the microsporidia, in particular the central repeating region differs significantly between the various Encephalitozoonidae (MicrosporidiaDb.org). This region has been suggested to function as an immunological masking region during infection, but there are no experimental data to support this hypothesis (Xu and Weiss, 2005; Bouzahzah et al., 2010; Weiss et al., 2014). The C and N terminal regions have more conservation, especially with regard to cysteine content (the presence of disulfide bridges in the assembly of the PT is supported by the ability of DTT and other reducing agents to solubilize the tube).

Four additional PTPs (PTP2 through PTP5) have been identified and characterized using proteomic and antibody-based approaches, and proteomic data suggests that there are additional PTPs in the PT (Peuvel et al., 2002; Bouzahzah et al., 2010; Weiss and Becnel, 2014; Weiss et al., 2014; Han et al., 2017, 2019). PTP2 is found at the same genomic locus as PTP1. The PTP2 from various microsporidia are more conserved in their properties such as molecular weight, basic isoelectric point (pI), high lysine content and cysteine residues when compared with PTP1 conservation (Delbac et al., 2001). PTP3 was found to be solubilized in the presence of SDS without adding a reducing reagent such as DTT, indicating it is not involved in disulfide bonding with other PTPs. It has also been suggested that PTP3 might be a scaffolding protein that plays an important role during the formation of the PT by interaction with other PTPs (Peuvel et al., 2002; Bouzahzah et al., 2010). When cross linking agents are used, a complex containing PTP1, PTP2, and PTP3 is obtained from intact PTs, indicating that these proteins do indeed interact (Peuvel et al., 2002; Bouzahzah et al., 2010). Similar to the genomic locus of PTP1/PTP2, the genes of PTP4 and PTP5 were also found to cluster together in many microsporidia genomes (Weiss et al., 2014).

A PTP4 monoclonal antibody which only stained the extruded tip of PT was identified, suggesting that a specific epitope of PTP4 could be important during the interaction of the PT with its host cell (Han et al., 2017). Using an immunoprecipitation assay followed by proteomic analysis a host cell receptor protein [Transferrin 1 (TfR1)] was identified that interacted with PTP4 (Han et al., 2017). In addition, it was found that PTP4 interacted with TfR-1 in the invasion synapse and that interference with the association of PTP4 and TfR-1 decreased the ability of *E. hellem* to invade its mammalian host cell (Han et al., 2017). As the sporoplasm forms a droplet at the tip of PT during germination we hypothesized that PTPs might be able to interact with sporoplasm proteins during the process of invasion. This concept is supported by the finding that a recently identified sporoplasm surface protein (SSP1) from *E. hellem* interacts with PTP4 in a yeast-two hybrid assay (Han et al., 2019).

## Observations on the Sporoplasm

During infection of host cells by microsporidia, the infectious sporoplasm is transported from spores via the PT, resulting in the transmission of the infection (Takvorian et al., 2005; Vavra and Larsson, 2014). During this process, the sporoplasm flows through the PT, appears as a droplet at the distal end of the PT and remains attached to the PT for several minutes (Korke, 1916; Ohshima, 1937; Gibbs, 1953; Lom, 1972; Weidner, 1972; Frixione et al., 1992; Han et al., 2017). It is likely that the sporoplasm interacts with the host cell within the protected environment of the invasion synapse during invasion. After the entrance of a sporoplasm into the host cell, it starts a reproduction cycle which includes meronts (proliferative forms), sporonts, sporoblasts, and terminates with the mature spores (Cali and Takvorian, 2014; Han and Weiss, 2017). The sporoplasm is tightly associated with the PT throughout spore germination and host cell invasion (Cali and Takvorian, 2014). The sporoplasm is very sensitive to osmotic stress and the formation of the invasion synapse is probably critical to its survival when it exits the PT (Weiss et al., 2014; Han et al., 2017).

Purification of the microsporidial sporoplasm has been very difficult and, up till now, only a few proteins have been localized and characterized in the sporoplasm plasma membrane. An ATP-binding cassette (ABC) transporter subfamily protein NoboABCG1.1 was identified from silkworm pathogen *Nosema bombycis*, the IFA and IEM analysis showed that NoboABCG1.1 is a membrane protein that is located on the sporoplasm, meront, and mature spore. Knocking down NoboABCG1.1 using an RNAi approach leads to a significant reduction in the growth of *Nosema bombycis* suggesting that this transporter was important for acquisition of essential nutrients for this organism (He et al., 2019). Four nucleotide transport proteins (NTT1-4) have been identified from other species of microsporidia (*Encephalitozoon cuniculi* and *Trachipleistophora hominis*) which were believed to be obtained from bacteria by horizontal gene transfer during the microsporidia evolution (Tsaousis et al., 2008; Heinz et al., 2014; Dean et al., 2018). Three of these NTTs have been shown to be in the sporoplasm membrane and all of these NTTs were demonstrated to be able to transport ATP, GTP, NAD^+^, and purine nucleotides from the host cytoplasm (Tsaousis et al., 2008; Heinz et al., 2014; Dean et al., 2018). The microsporidia have a highly reduced genome which contains ∼3000 protein coding genes, they lack functional mitochondria, and lack almost all of the genes for ATP generation other than glycolysis, therefore, these NTTs which are expressed on the parasite surface are thought to be critical strategies for microsporidia to acquire ATP and other purine nucleotides for energy and biosynthesis from their host (Katinka et al., 2001; Keeling et al., 2010; Heinz et al., 2012; Dean et al., 2016). A recent study demonstrated that another sporoplasm surface located protein family, the Microsporidia major facilitator superfamily (MFS) transport proteins are used as a second set of transporters to acquire energy and nucleotides from host cells. Four MFS proteins were identified from *Trachipleistophora hominis* (ThMFS1-4) and ThMFS1 and ThMFS3 were demonstrated to be located in the sporoplasm plasma membrane during infection (3 to 96 h post infection in cell culture). Further study revealed that all four ThMFS can transport ATP, GTP, and purine; thus they have a similar function to the NTTs (Major et al., 2019). However, neither NTTs nor ThMFS can transport the pyrimidine nucleotides suggesting that microsporidia have a yet unknown pyrimidine nucleotide import system (Heinz et al., 2014; Dean et al., 2018; Major et al., 2019).

While microsporidia were originally believed to not have mitochondria, it has been discovered that they have a highly reduced mitochondria termed a mitosome that has lost its mitochondrial genome and capacity for ATP generation (Williams et al., 2002; Goldberg et al., 2008). Mitosomes are double-membrane-bounded organelles which have been found in several species of parasites such as Microsporidia, Diplomonads, Amoebozoa, and Apicomplexa (Tovar et al., 1999, 2003; Williams et al., 2002; Keithly et al., 2005). Compared to mitochondria the mitosomes are morphologically smaller, lack cristae, and lack their own DNA (making them completely reliant on importing nuclear encoded proteins for their functions and organelle maintenance) (Burri et al., 2006; Hans-Peter Braun, 2009; Tachezy, 2019). Microsporidian mitosomes have lost their capacity for ATP production through oxidative phosphorylation, even though they can use glycolysis for energy generation, but this pathway, while active in spores, appears to not be active during the stage of intracellular growth and replication inside of host cytosol (Dolgikh et al., 2011; Heinz et al., 2012; Williams et al., 2014). Microsporidia can use glycolysis for energy generation, but this pathway, while active in spores, appears to not be active during the stage of intracellular growth and replication inside of the host cytosol (Dolgikh et al., 2011; Heinz et al., 2012; Williams et al., 2014). Thus, microsporidia depend on their host cells for energy and mitochondria accumulate around the microsporidia [this is clearly observable in Encephalitozoonidae residing in a parasitophorous vacuole within their host cells (Han et al., 2019)].

The molecular mechanism of mitochondria and microsporidia association is still unknown. A recent study revealed that *E. hellem* sporoplasm surface protein 1 (EhSSP1), a protein expressed on the surface of the sporoplasm, is involved in the interaction of microsporidia with host cell mitochondria. EhSSP1 was demonstrated to interact with all three forms of voltage-dependent anion selective channels (VDAC1-3), which are mainly expressed in the cytoplasm of the outer mitochondrial membrane. Inhibiting this interaction decreased the association of mitochondria with the microsporidian parasitophorous vacuole (Han et al., 2019). Interaction of EhSSP1 with VDAC probably facilitates energy acquisition by the microsporidia in its host cell (Han et al., 2019). Interestingly, EhSSP1 also interacted with an unidentified host cell protein in the invasion synapse, and might also have another role during invasion.

## Microsporidia Invasion

**FIGURE 5:**
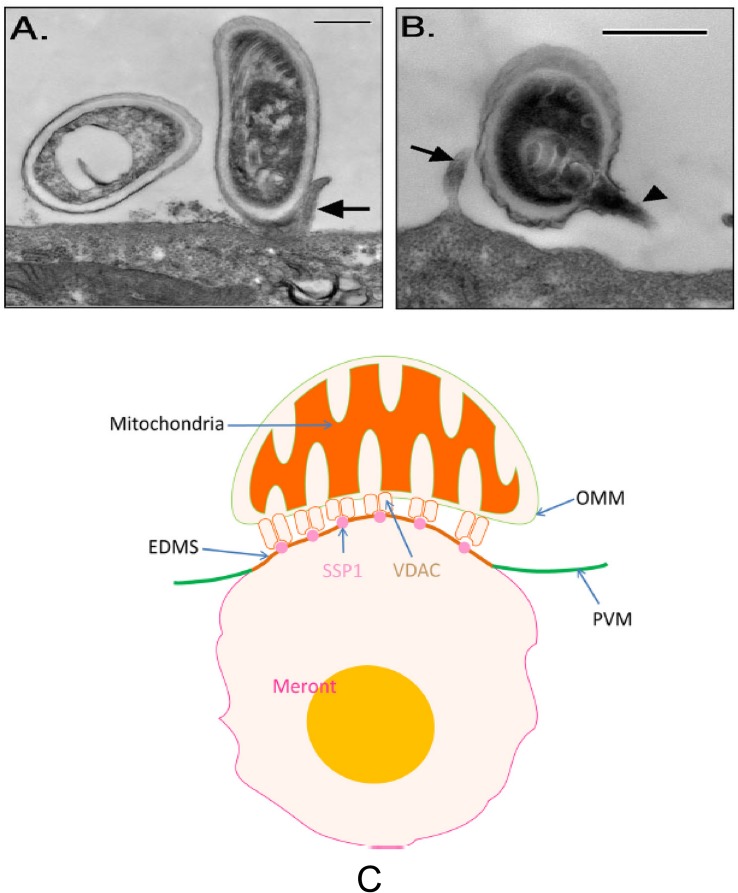
Transmission electron microscopy of *E. intestinalis* spores attached to Caco-2 cell surfaces. The attached spores appear to be in direct contact with the cell surface or microvilli and have either intact, unextruded polar filaments **(A)** or extruded PTs **(B).** Bar is 500 nm. Arrows show host cell microvilli, and the arrowhead points to the *E. intestinalis* PT. Reprinted with permission of the author (JR Hayman) and the publisher (Hayman et al., 2005). **(C)** A model of the binding of mitochondria with the parasitophorous vacuole via the interaction of VDAC on the out mitochondria membrane with SSP1 on the surface of meronts. Reprinted with permission of the publisher (Han et al., 2019).

Microsporidia infection of host cells involves the rapid extrusion of the PT and transfer of the sporoplasm into the host cell (Weidner, 1972; Frixione et al., 1992; Takvorian et al., 2005; Han et al., 2017). Generally, the adherence of microsporidian spores to host cells or to the vicinity of the host cells is the first step in the infection process (Weidner, 1972; Han et al., 2017). Spore wall proteins SWPs probably play a crucial role during the interaction of microsporidia and host cells (Southern et al., 2007). Several SWPs which can interact with host cells by binding to the heparin-binding motif (HBM) and host cell surface sulfated glycosaminoglycans (GAGs) have been identified from *Nosema bombycis, Encephalitozoon spp*., and *Antonospora locustae* (Hayman et al., 2001; Hayman et al., 2005; Southern et al., 2007; Li et al., 2009; Wu et al., 2009; Chen et al., 2017). Besides the interaction of HBM with GAGs during spore adherence to host cells, a separate study reported that host cell integrin is also involved in *E. intestinalis* adherence and infection of its host cells (Leonard and Hayman, 2017). Analysis of the *E. intestinalis* genome demonstrated numerous hypothetical proteins that were predicted to contain the canonical integrin-binding motif arginine-glycine-aspartic acid (RGD), which is the binding motif involved in the interaction of extracellular matrix (ECM) proteins with host cell integrins. Proteins that interact with host cell integrins have been found in many pathogenic microbes that adhere to host cells including viruses, bacteria and parasites (Patti et al., 1994; Finlay and Falkow, 1997; Rostand and Esko, 1997; Bartlett and Park, 2010). Incubation of host cells with RGD-peptides or recombinant alpha3 beta1 and alpha 5 beta 1 human integrin proteins inhibited microsporidia spore adherence and host cell infection (Leonard and Hayman, 2017). This suggests that spore adherence is important in the germination and subsequent invasion of host cells (Figure 5).

In addition to binding to GAGs, analysis of NbSWP26 from *Nosema bombycis* also demonstrated that it could interact with the turtle-like protein (BmTLP) of the silkworm *Bombyx mori* (Zhu et al., 2013). BmTLP is a lgSF member protein which is a cytokine receptor, cell surface antigen receptor and cell adhesion molecules that are involved in antigen presentation to vertebrate lymphocytes, co-receptors and co-stimulatory molecules of the immune system (Barclay, 2003). This interaction of NbSWP26 with BmTLP suggests that it might act as a receptor that facilitates spore invasion of silkworm cells (Zhu et al., 2013).

It has been shown that attached spores (Figure 5) may be phagocytosed by both professional and non-professional phagocytes via an actin based mechanism (Weidner and Sibley, 1985; Couzinet et al., 2000; Foucault and Drancourt, 2000; Hayman et al., 2005; Leitch et al., 2005). Interestingly, NbSWP5 from *Nosema bombycis* can protect spores from phagocytic uptake by cultured insect cells revealing that it may function both for structural integrity and in modulating host cell invasion (Cai et al., 2011). Phagocytosed spores will be transferred to endosomal and eventually to lysosomal compartments; however, phagocytosed spores have been shown to germinate resulting in infection of either the host cell that phagocytized the spore or adjacent cells (Franzen, 2004, 2005; Franzen et al., 2005).

The interaction of PT and sporoplasm with host cell during microsporidia infection is not fully understood. After germination of the polar tube, PTP1 (a mannosylated protein with a significant number of O-linked mannosylation modification sites) can interact with mannose binding receptors on the host cell surface, thereby, attaching the PT to the host cell (Xu et al., 2003, 2004). As the PT pushes into the host cell it creates an invagination in the host cell creating a microenvironment which we have termed the invasion synapse (Figure 3). Within this protected environment the sporoplasm exits the PT, it is not known if the PT penetrates the host cell membrane delivering the sporoplasm into the host cytosol or if the sporoplasm penetrates directly into the host cell within this invasion synapse. For microsporidia that reside in a parasitophorous vacuole we believe, based on our published data (Han et al., 2017, 2019), that the second hypothesis is probable and that interactions of PTPs and the sporoplasm membrane with the host cell membrane are important during invasion. To this end, polar tube protein 4 (PTP4) has been demonstrated to have a specific epitope on the tip of the PT and this epitope was shown to interact with host cell transferrin receptor (TfR1) (Han et al., 2017). TfR1 is the main receptor for most cells that take up iron and is involved in iron uptake via cathrin-mediated endocytosis (Qian et al., 2002). Several viruses have been demonstrated to utilize the TfR1 pathway for binding and subsequent invasion of their host cells. The PTP4 TfR1 interaction may trigger the clathrin-mediated endocytosis pathway and could help to facilitate the process of invasion within the invasion synapse (Han et al., 2017).

After the sporoplasm invades or is transported into the host cell cytoplasm, it enters the proliferative phase of the life cycle marked by extensive multiplication via merogony. The location of this developmental stage within the host cell varies by genus (Cali and Takvorian, 2014); it can occur either in direct contact with the host cell cytoplasm (e.g., *Nosema, Enterocytozoon)*, in a parasitophorous vacuole lined by a host-produced single membrane (e.g., *Encephalitozoon*), in a parasite-secreted amorphous coat (e.g., *Pleistophora, Trachipleistophora, Thelohania)*, or surrounded by endoplasmic reticulum of the host (e.g., *Endoreticulatus, Vittaforma*) (Sprague et al., 1992; Martinez et al., 1993; Bigliardi and Sacchi, 2001; Cali and Takvorian, 2003, 2014). The interactions of various microsporidial developmental stage-specific surface proteins with host cell cytoplasm proteins or organelles (e.g., mitochondria and endoplasmic reticulum) during the process described above remains to be determined.

Host VDACs have been shown to be concentrated at the interface of host cell mitochondrial and microsporidia parasitophorous vacuole membrane (PVM) (Hacker et al., 2014). The function of VDACs which mainly locate at the outer membrane of mitochondria as channel proteins is to control the movement of adenine nucleotides, NADH, and other metabolites across the membrane (Blachly-Dyson and Forte, 2001; Cesura et al., 2003; Rostovtseva et al., 2005). The association of VDACs to the PVM has been hypothesized to be a strategy used by microsporidia to maximize its ATP supply from its host cells (Hacker et al., 2014). However, the interaction target of VDACs in microsporidia was not identified until recently when EhSSP1 was identified from *Encephalitozoon hellem* (Han et al., 2019). Studies of EhSSP1 demonstrated that the microsporidia tether the host mitochondria to its PVM during intracellular development by hijacking VDACs using EhSSP1, which is probably critical for energy uptake by the replicative forms of this organism (Han et al., 2019) (Figure 5).

After replication, many microsporidia appear to exit the host cell by lysis and/or apoptosis of the infected cell, however, in cell culture and in some animal models one can see adjacent foci of infection suggestive of cell to cell spread of these pathogens (Weiss and Becnel, 2014; Balla et al., 2016). There has been very limited study on the molecular pathways which provide the major modes for egress of microsporidia from host cells. A study on *Nematocida parisii* has shown that microsporidia escape from intestinal cells by co-opting the host vesicle trafficking system and escaping into the lumen (Szumowski et al., 2014). A host small GTPase protein called RAB-11, which apically localizes in many polarized epithelial cells was required for spore-containing compartments to fuse with the apical plasma membrane and direct microsporidian exocytosis (Szumowski et al., 2014). Moreover, during the process of exiting, an intestinal-specific isoform of *C. elegans* actin-5 can form coats around the membrane compartments which contain the exocytosing spores after fusion with the apical membrane and the smGTPases rab-5, rab-11, cdc-42, and ced-10/Rac 1 promote the formation of actin coats during this process (Szumowski et al., 2016).

## Conclusion

Microsporidia are opportunistic pathogens of immune suppressed patients and the clinical spectrum of diseases they cause is expanding with the introduction of new immune modulatory therapies. Furthermore, they are important pathogens of economically important insects and animals. The mechanism of invasion used by these pathogens is unique with a highly specialized invasion apparatus which despite its description over 125 years ago is still not understood. Progress, however, has been made in understanding the proteins in this invasion apparatus and the interaction of these proteins with some host cell proteins. Nonetheless, the mechanism of how microsporidia enter host cells and establish host pathogen relationships seen in the various microsporidia species has not been determined. In addition, the egress of microsporidia from its host cell when it has completed its replicative cycle is another area that deserves study. Understanding how microsporidia use host cell proteins in both invasion and egress will provide insight into their impact on hosts and enhance our current understanding of the transmission dynamics of this pathogen. In addition, understanding these processes will provide information needed for new therapeutic approaches to control these pathogenic protists.

## Author Contributions

BH, PT, and LW composed the manuscript, compiled information from the literature, and designed the figures and tables. LW edited the final manuscript.

## Conflict of Interest

The authors declare that the research was conducted in the absence of any commercial or financial relationships that could be construed as a potential conflict of interest.
